# Supplementary figures and images for: Automatic target validation based on neuroscientific literature mining for tractography
Source: Front Neuroanat. 2015 May 27;9:66. doi: 10.3389/fnana.2015.00066 (PMC4445321; doi:10.3389/fnana.2015.00066)

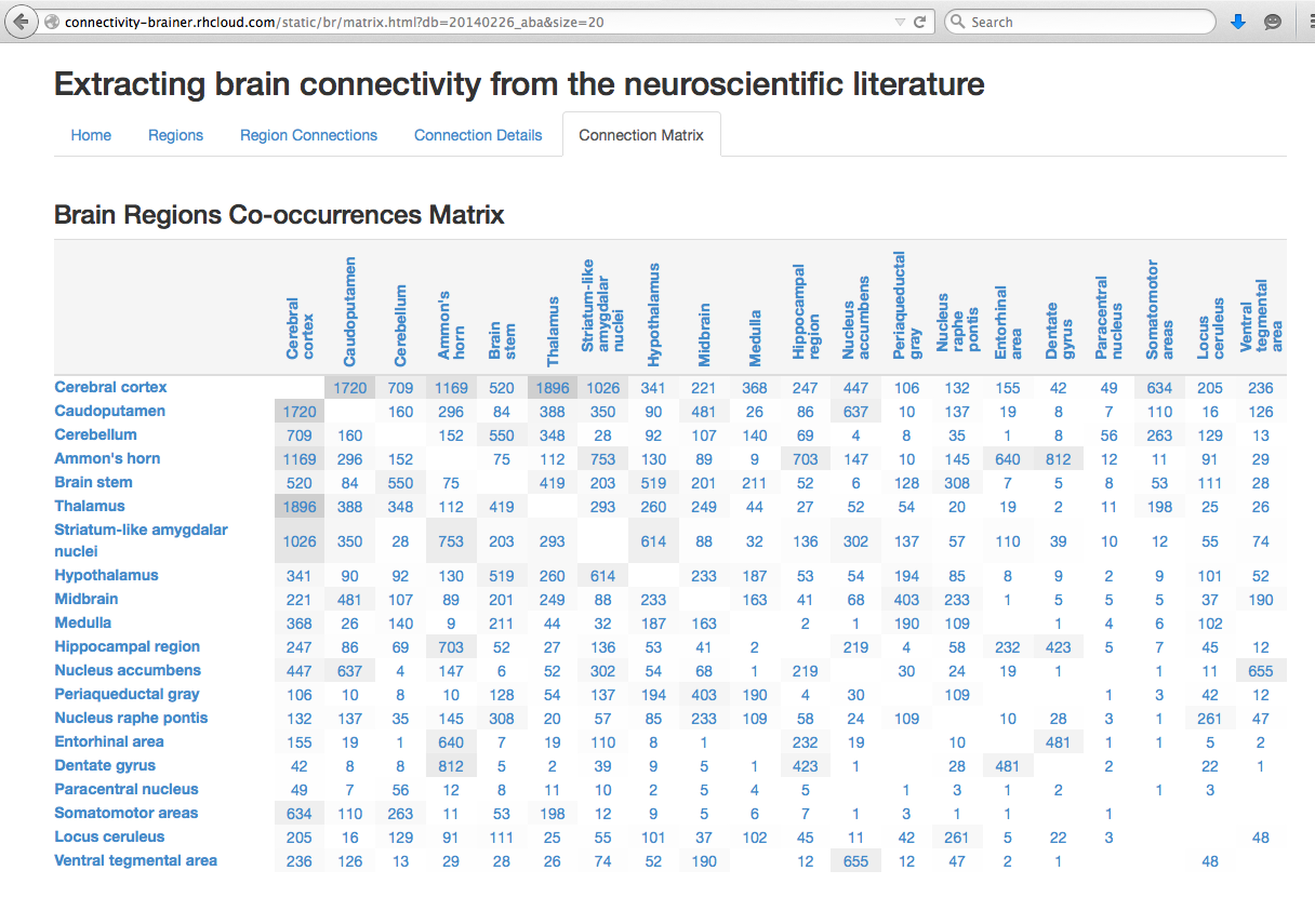

Supplement: Supplementary Figure 1 — Brain regions co-occurrences matrix displaying the top 20 regions for which the most connection mentions was found. Matrix values represent the number of connectivity events, normalized by the confidence that each event has been extracted correctly (precision). All matrix values are linked to the corresponding detailed list of article sentences (see Supplementary Figure 3). The corresponding url for that figure is http://connectivity-brainer.rhcloud.com/static/br/matrix.html?db=20140226_aba&size=20. [file Image1.JPEG]

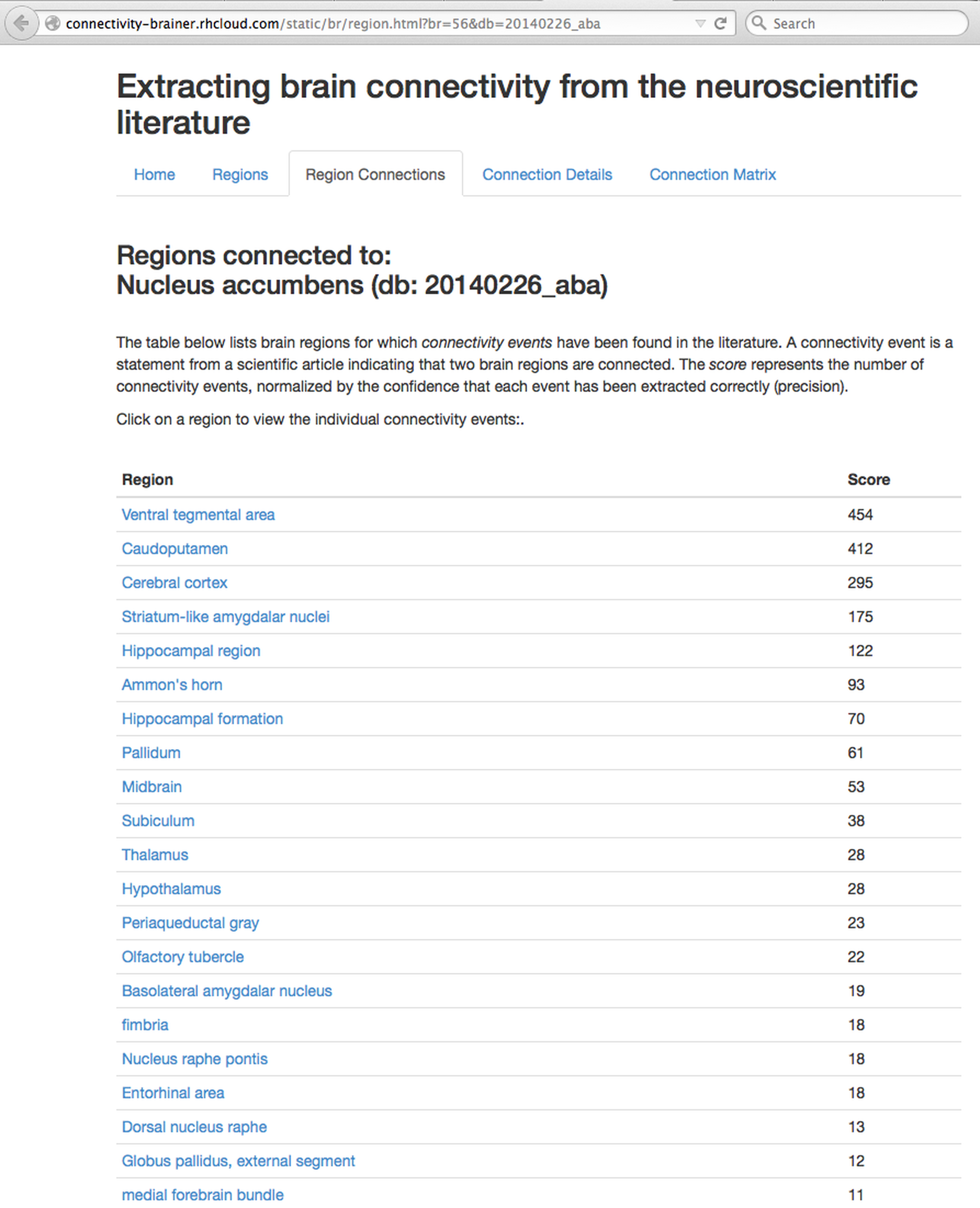

Supplement: Supplementary Figure 2 — Listing of brain regions potentially connected to Nucleus accumbens, for which connectivity events have been found in the literature. The score represents the number of connectivity events, normalized by the confidence that each event has been extracted correctly (precision). All regions are linked to the corresponding detailed list of article sentences (see Supplementary Figure 3). The corresponding url for that figure is http://connectivity-brainer.rhcloud.com/static/br/region.html?br=56&db=20140226_aba. [file Image2.JPEG]

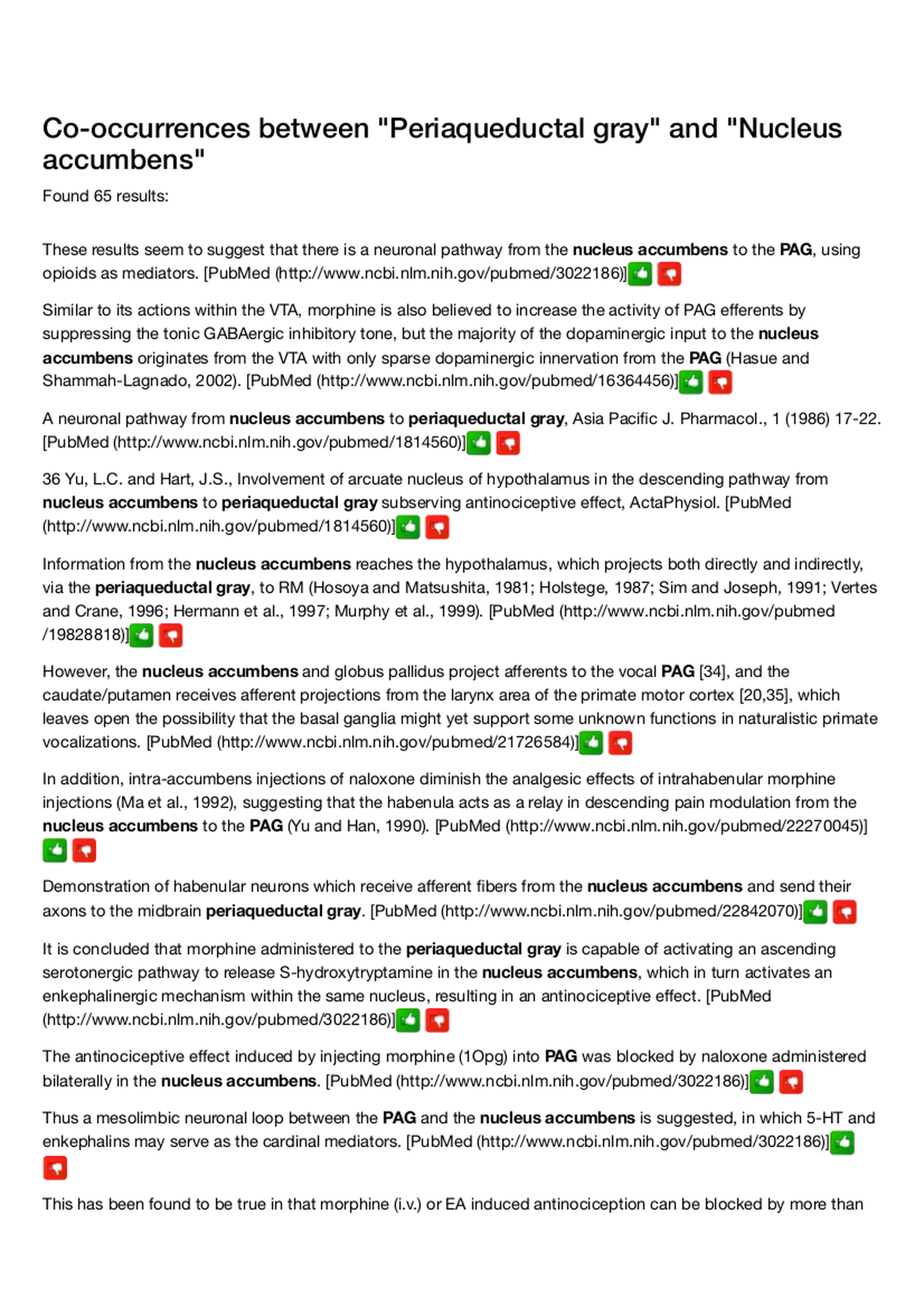

Supplement: Supplementary Figure 3 — Detailed list of sentences from neuroscientific articles, in this case between “Periaqueductal gray” and “Nucleus accumbens” (list truncated for readability). Each sentence is linked to the original article on PubMed. Additionaly, the user has the ability to provide feedback: clicking on the red icon (thumbs down) will remove that sentence, and log it into the database. Similarly, clicking on the green icon (thumbs up) will confirm that sentence and log it in the database. The corresponding url for that figure is http://connectivity-brainer.rhcloud.com/static/br/details.html?br1=795&br2=56&db=20140226_aba/. [file Image3.JPEG]
